# Systematized delusions in a patient with covert hepatic encephalopathy: A clinicopathological insight into prodromal dementia with Lewy bodies

**DOI:** 10.1002/pcn5.70247

**Published:** 2025-11-11

**Authors:** Hiroshige Fujishiro, Ito Kawakami, Kenichi Oshima, Youta Torii, Shusei Arafuka, Shuji Iritani, Kenji Ikeda

**Affiliations:** ^1^ Department of Psychiatry Nagoya University Graduate School of Medicine Nagoya Aichi Japan; ^2^ Dementia Research Project Tokyo Metropolitan Institute of Medical Science Tokyo Japan; ^3^ Department of Psychiatry Tokyo Metropolitan Matsuzawa Hospital Tokyo Japan; ^4^ Department of Psychiatry Okehazama Hospital Fujita Mental Care Center Toyoake Aichi Japan

**Keywords:** delirium, DLB, Lewy body disease, parkinson disease, psychosis

## Abstract

**Background:**

Late‐onset psychosis is an early clinical manifestation of psychiatric‐onset prodromal dementia with Lewy bodies (DLB); however, its underlying neuropathology remains poorly understood. Clinicopathological correlations are often limited by the gap between symptom onset and the autopsy.

**Case Presentation:**

A 66‐year‐old man with autopsy‐confirmed DLB presented with persistent systematized delusions. After treatment for liver cirrhosis during hospitalization, the patient's physical symptoms improved; however, persecutory delusions developed. The patient was clinically diagnosed with covert hepatic encephalopathy (HE). The delusions were atypical for covert HE, suspecting delusional disorder. His systematized delusions persisted for 3 months until his death, without the development of cognitive decline or Parkinsonism during his lifetime. An autopsy revealed an early transitional type of Lewy body disease with minimal Alzheimer's type II astrocytes indicative of HE. Severe neuronal loss was observed in the locus coeruleus (LC), while the substantia nigra (SN) and nucleus basalis of Meynert (nbM) were preserved. Abundant alpha‐synuclein‐positive structures were identified in the LC, periaqueductal gray matter, nbM, amygdala, and thalamus, with sparse involvement of the SN, neocortex, peripheral autonomic nervous system, including the heart and gastrointestinal tract.

**Conclusion:**

Selective Lewy body involvement, sparing the SN and neocortex, may explain the isolated psychiatric symptoms in the absence of Parkinsonism or dementia. Systemic conditions such as covert HE may have contributed to the emergence of persistent delusions. This case highlights the need for multidisciplinary approaches that integrate psychosomatic assessments with neuropathological investigations to evaluate late‐onset psychosis.

## BACKGROUND

Several clinicopathological studies have revealed that isolated psychiatric symptoms can precede the onset of dementia with Lewy bodies (DLB) by years or even decades.^1^, ^2^, ^3^, ^4^ However, long‐term clinical courses have suggested the need to consider additional pathologies that may emerge in the later stages of the disease up to the time of death. This suggests that a delay between symptom onset and autopsy may limit clinicopathological correlations regarding the underlying pathology of the initial psychiatric manifestations in cases of psychiatric‐onset prodromal DLB.^5^

This study reports the case of a 66‐year‐old man with autopsy‐confirmed DLB who exhibited late‐onset systematized delusions. Clinically, the condition was classified as covert hepatic encephalopathy (HE) based on the criteria of the International Society for Hepatic Encephalopathy and Nitrogen Metabolism (ISHEN).^6^ Persistent systematized delusions are atypical for covert HE, which raises the suspicion of a delusional disorder.^7^ Notably, progressive cognitive decline and Parkinsonism were not observed during the patient's lifetime. The interval between the onset of psychiatric symptoms and autopsy was only 3 months. In the context of early phase prodromal DLB, the potential involvement of Lewy body (LB) pathology was discussed in the emergence of systematized delusions. Informed consent was obtained from the patient's family for the publication of this report, and all efforts were made to preserve patient anonymity.

## CASE PRESENTATION

A 66‐year‐old man with chief complaints of dyspnea and fatigue was admitted to the internal medicine ward in late August owing to liver cirrhosis and ascites. He had been employed before hospitalization. He had no medical history, including psychiatric disease such as alcohol use disorder.^7^ His physical symptoms improved with treatment; however, in late September, he developed persecutory delusions, believing that hospital staff were attempting to poison him under orders from North Korea. Owing to his refusal of treatment, continued hospitalization became difficult, and the patient was discharged.

Following a 1‐month hospitalization, the patient's delusions persisted. He claimed, “I infiltrated the hospital to investigate at my own expense. The hospital tried to kill me under North Korea's control,” and “My house is wiretapped.” He repeatedly contacted the police and reported that he was at risk of assassination by North Korean agents. Despite these fixed delusions, he was functionally capable during a 3‐week period at home, preparing documents to apply for public assistance after losing his job. While visiting a welfare center to submit documents, he complained of dyspnea. The concerned staff member called an ambulance, and the patient was re‐hospitalized for liver cirrhosis with pleural effusion.

**Figure 1:**
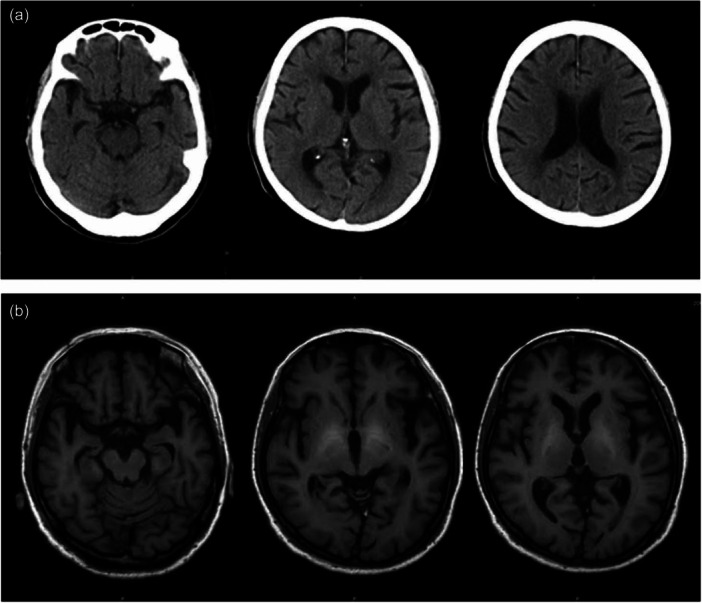
Morphologic brain imaging. Head Computed Tomography was unremarkable at admission to the psychiatric hospital (a). T1‐weighted brain magnetic resonance imaging revealed hyperintensity in the bilateral basal ganglia when overt hepatic encephalopathy was diagnosed (b).

Persecutory delusions made continued hospitalization difficult. In late October, the patient was transferred to the psychiatric ward of a general hospital as the facility lacked psychiatric services. Upon admission, behavioral disturbances related to delusions were observed; however, the patient remained oriented toward time and place and showed no signs of asterixis. Computed tomography of the head revealed no remarkable findings (Figure 1a). Elevated serum ammonia levels (96 μg/dL), along with diuretic use and constipation, were identified as potential precipitating factors for HE. According to the ISHEN consensus,^6^ the absence of disorientation and asterixis indicated that his condition corresponded to covert HE. Systematized delusions are atypical for covert HE, warranting the inclusion of primary psychiatric disorders, such as delusional disorders, in the differential diagnosis. Physical treatment was continued, along with haloperidol administration. Nevertheless, he persistently claimed that North Korea targeted him because he had witnessed a secret during his initial hospitalization. He further asserted that he had died because of inappropriate treatment. Laboratory evaluations, including tests for hepatitis B, hepatitis C, and autoimmune markers, suggested that the liver cirrhosis was most likely attributable to nonalcoholic steatohepatitis.

Three weeks post‐admission to the psychiatric ward, he developed fluctuating levels of consciousness and mild asterixis. T1‐weighted brain magnetic resonance imaging (MRI) revealed hyperintensities in the bilateral basal ganglia (Figure 1b), leading to a clinical diagnosis of overt HE. One month post‐diagnosis, the patient died of respiratory failure. In the final month of his life, he reported transient visual hallucinations of the God of death, and his delusions regarding North Korea remained unchanged. He expressed remorse and apologized to the attending doctor for becoming involved in what he perceived as a conflict with North Korea. The total duration of persistent delusions was approximately 3 months. Notably, he did not develop progressive cognitive decline or Parkinsonism throughout his lifetime.

### Pathological findings

**Figure 2:**
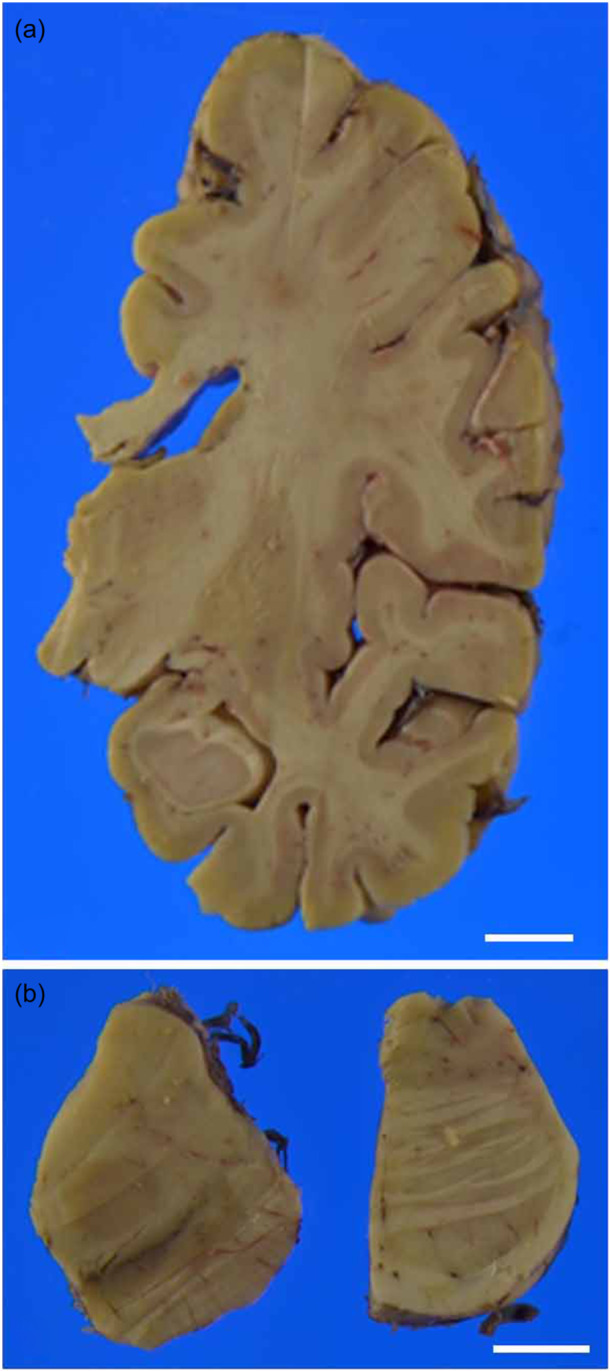
Macroscopic finding. A coronal section at the level of the hippocampus showed no atrophy or discoloration (a). Transverse sections of the midbrain showed well‐preserved pigmentation of the substantia nigra, while there was a loss of pigmentation in the locus coeruleus (b). Bar 10 mm.

**Figure 3:**
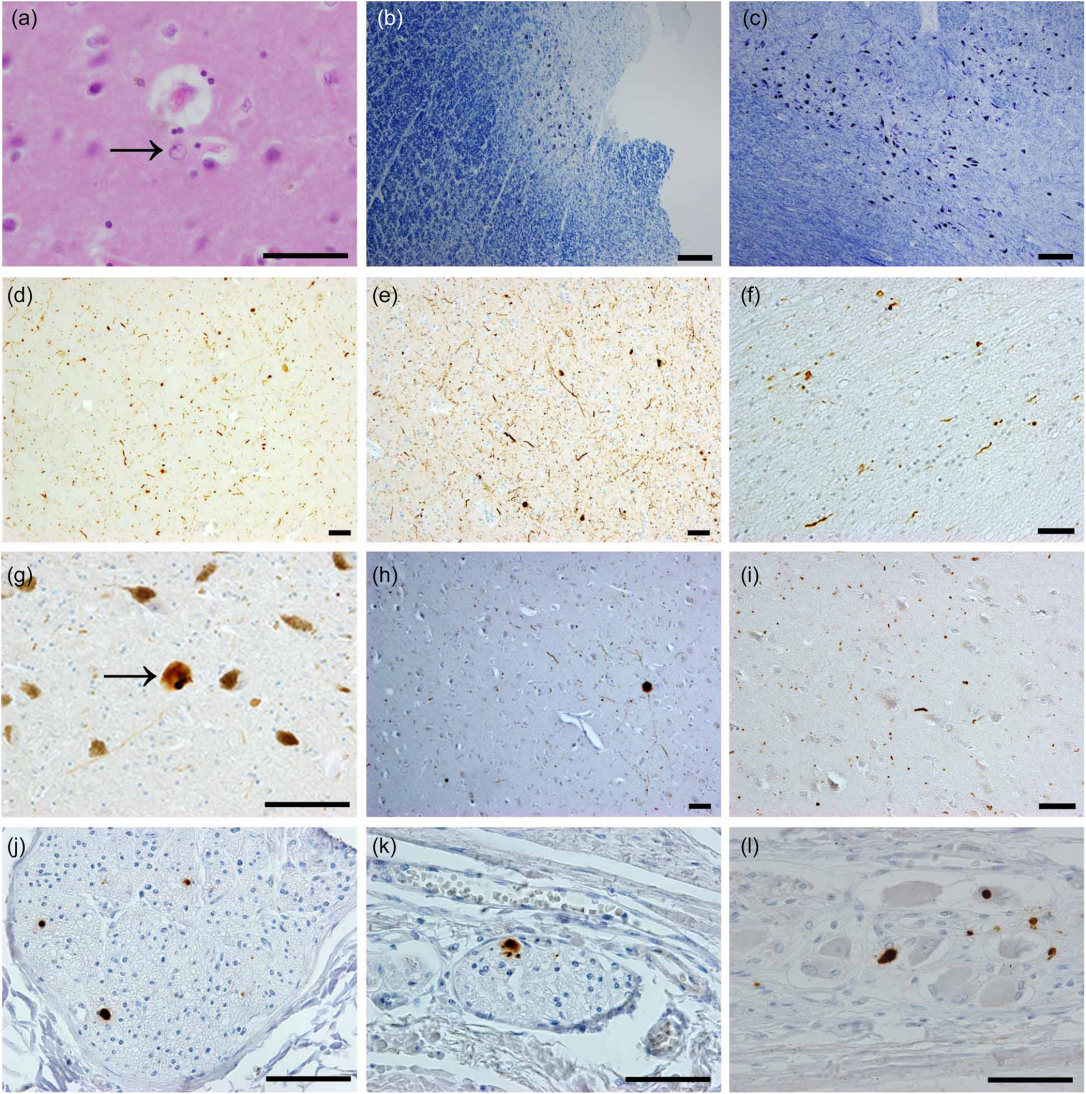
Microscopic finding. Alzheimer type II astrocyte in the basal ganglia was identified on hematoxylin–eosin (H&E) staining (a). On Klüver‐Barrera staining, moderate neuronal loss with Lewy bodies was observed in the locus coeruleus (b), while abundant neurons in the substantia nigra (SN) were identified (c). Alpha‐synuclein immunostaining revealed abundant positive structures in the nucleus basalis of Meynert (d) and amygdala (e). Sparse α‐synuclein‐immunoreactive structures in the olfactory bulb (f), SN (g), cingulate cortex (h), and temporal neocortex (i). There were sparse α‐synuclein‐immunoreactive structures in the peripheral autonomic nervous system, including the sympathetic fibers in the anterior wall of the left ventricle of the heart (j), the myenteric and submucosal plexuses from the esophagus (k), and the stomach (l). Bar 50 μm (a, e, f, g, h, i, j, k, l), 200 μm (b, c, d).

The macroscopic findings were unremarkable, except for invisible pigmentation in the locus ceruleus (LC) (Figure 2). The pigmentation of the substantia nigra (SN) was well‐preserved. Microscopic examination of hematoxylin and eosin (H&E)‐stained sections revealed Alzheimer type II astrocytes in the basal ganglia and neocortex (Figure 3a). These astrocytes were occasionally observed in coronal sections of the basal ganglia, with their number approaching the threshold of five type II astrocytes per 20 high‐power fields (HPFs, magnification ×400), which is considered characteristic of HE.^8^ In overt HE cases, a mean of 19.8 astrocytes per 20 HPFs has been reported.^8^

Klüver‐Barrera staining revealed vascular hyalinization accompanied by mild myelin pallor. Immunostaining for β‐amyloid demonstrated sparse, localized, diffuse plaques and amyloid angiopathy in the temporal gyrus. A small number of neurofibrillary tangles (NFTs) were observed in the hippocampus using both anti‐phosphorylated tau immunostaining and modified Gallyas‐Braak staining. According to Braak NFT staging, these findings correspond to stage I.^9^ Argyrophilic grains were absent. Based on the National Institute on Ageing‐Alzheimer's Association guidelines, these findings represent a low level of neuropathological change in Alzheimer's disease (A1B1C0).^10^

H&E staining also revealed the presence of LBs. The dorsal motor nucleus of the vagus (DMN) and LC exhibited moderate to severe neuronal loss, with some residual neurons containing LBs. In contrast, the SN displayed a preserved neuronal architecture and lacked typical LBs (Figure 3b,c). The nucleus basalis of Meynert (nbM) showed preserved neuronal density, although LBs were present. Alpha‐synuclein immunostaining revealed abundant immunoreactive structures, including both LBs and Lewy neurites in the DMN, LC, periaqueductal gray matter, nbM, amygdala, hypothalamus, and thalamus (Figure 3d,e). Sparse alpha‐synuclein‐positive structures were identified in the olfactory bulb, SN, nucleus accumbens, and neocortex (Figure 3f–i).

To minimize the possibility of false negatives in the peripheral nervous system, 33 sections were screened using alpha‐synuclein immunostaining. Sparse alpha‐synuclein‐immunoreactive structures were found in the anterior wall of the left ventricle (7 of 18 sections) and in the myenteric and submucosal plexuses of the esophagus and stomach (6 of 9 sections) (Figure 3j–l).^11^, ^12^ No such structures were detected in any of the three sections from the colon or the three from the adrenal glands.^13^ A semi‐quantitative assessment of LB pathology is summarized in Table 1. This distribution pattern was consistent with that of early transitional Lewy body disease (LBD), indicating a marginal DLB phenotype. Considering the low level of concurrent AD pathology, this patient was classified as having a high likelihood of DLB pathology. Neuronal loss in the SN was rated as “none,” consistent with the absence of Parkinsonism.^15^ No phosphorylated 43‐kDa TAR DNA‐binding protein (TDP‐43)‐positive structures were detected in the brain.

**Table 1:** Distribution of Lewy bodies.

Lewy body type pathology	Brainstem region			Basal forebrain/limbic regions				Neocortical regions		
	IX‐X	LC	SN	nbM	Amygdala	Transentorhinal	Cingulate	Temporal	Frontal	Parietal
Brainstem‐predominant LBD	1–3	1–3	1–3	0–2	0–2	0–1	0–1	0	0	0
Our case	2	2	0‐1	2	2	0–1	0–1	0–1	0–1	0
Kaneko's case^14^	2–3	2	2	0–1	2–3	2	0	0	0	0
Transitional (Limbic) LBD	1–3	1–3	1–3	2–3	2–3	1–3	1–3	0–2	0–1	0
Diffuse neocortical LBD	1–3	1–3	1–3	2–3	3–4	2–4	2–4	2–3	1–3	0–2

*Note*: The severity of Lewy body pathology was assessed semi‐quantitatively according to the pathological criteria of the Fourth Consortium for Dementia with Lewy Bodies.

Abbreviations: IX‐X, dorsal motor nucleus of the vagus; LBD, Lewy body disease; LC, locus coeruleus; SN, substantia nigra; nucleus basalis of Meynert.

## DISCUSSION

HE is clinically defined as brain dysfunction in patients with liver insufficiency or a portosystemic shunt. Its manifestations encompass a broad spectrum of neurological and psychiatric abnormalities ranging from subclinical changes to coma. The West Haven criteria, which classify HE into four grades (I–IV), are widely used for clinical assessment.^6^ According to this system, overt HE is diagnosed when the condition exceeds grade II and is characterized by at least temporal disorientation and/or the presence of asterixis. Based on these criteria, our patient was classified as having covert HE (grade ≤ I) during the initial 2 months following the onset of delusions, and as having overt HE (grade ≥ II) during the final month preceding death. As a characteristic of Grade I according to the West Haven criteria (covert HE),^6^ impaired attention and subtle personality changes are listed, suggesting that the presence of persistent systematized delusions may be atypical in this early stage of HE.

HE is neuropathologically characterized by the presence of Alzheimer's type II astrocytes. The diagnostic specificity of this pathological hallmark has been recently investigated.^8^ In one study, Alzheimer type II astrocytes were observed not only in all 21 examined cases of HE but also in a wide range of patients without HE.^8^ The study demonstrated that while the presence of Alzheimer type II astrocytes supports the diagnosis of antemortem HE, diagnostic specificity increases with a higher number of these cells in the basal ganglia.^8^ In our patient, the neuropathological examination revealed a relatively small number of Alzheimer's type II astrocytes in the basal ganglia, which is consistent with mild HE in terms of pathological severity. These clinicopathological findings suggest that the persistent systematized delusions observed at the time of the first hospitalization may have been associated with factors other than covert HE.

The Consortium on DLB criteria recommends that the neuropathological diagnosis be expressed as a probability statement, reflecting the likelihood that the distribution of LB pathology correlates with a premortem clinical diagnosis of DLB.^15^ During the covert HE period following the initial admission to the internal medicine ward, symptoms such as constipation and systematized delusions were observed, serving as supportive features of prodromal DLB.^5^ In the overt HE period during the final month preceding death, the patient exhibited transient visual hallucinations and fluctuations in attention and alertness, resembling episodes of delirium.^16^ While these manifestations may represent core features of prodromal DLB, they also overlap with the clinical picture of overt HE. The marginal‐type DLB pathology observed in this patient was consistent with the absence of full premortem DLB syndrome, including dementia. Additionally, the minimal involvement of LB pathology in the SN corresponds to a lack of Parkinsonism. Kaneko et al. also reported an autopsy case of marginal‐type DLB that developed forgetfulness after initiating repeated chemotherapy for lymphoma (Table 1).^14^ During his lifetime, he exhibited transient visual hallucinations, but no Parkinsonism, which was consistent with preservation of the SN. We reported an autopsy case of delirium‐onset prodromal DLB in which the patient exhibited transient visual hallucinations during pharmacological treatment for heart failure.^17^ Overt cognitive decline was not identified until the onset of a second delirium episode due to HE during the final 3 months preceding death. An autopsy performed approximately 1 year after the first delirium episode revealed transitional LBD. The probability‐based classification scheme for the pathological diagnosis of DLB suggests susceptibility to systemic conditions such as covert HE, lymphoma, and heart failure, which may trigger delirium‐like episodes, including transient visual hallucinations and attention deficits.^15^, ^16^ Thus, marginal‐type DLB may be associated not only with late‐onset psychosis but also with clinical phenotypes characterized by a delirium‐onset presentation in terms of overlaps in clinical symptomatology.^5^, ^16^

Hawkes et al. originally proposed the dual‐hit hypothesis, suggesting that the pathological accumulation of alpha‐synuclein begins simultaneously in the olfactory bulb and enteric nervous system plexuses at the earliest stage of the disease.^18^ More recently, Horsager et al. introduced a hypothetical model distinguishing body‐first and brain‐first subtypes of LBD.^19^ In which the peripheral autonomic nervous system is the primary site of alpha‐synuclein pathology, with subsequent propagation to the central nervous system (CNS) via the vagus nerve or sympathetic pathways to the sympathetic trunk and heart. In contrast, the brain‐first subtype is characterized by initial alpha‐synuclein accumulation within the CNS without prior involvement of the peripheral autonomic nervous system. The same group identified the amygdala as the most probable initial site of brain‐first pathology, followed by the SN and LC.^20^ In this case, early involvement of LB pathology in both the peripheral and central nervous systems suggests a simultaneous onset rather than the sequential pattern proposed by Horsager et al. Minimal LB pathology in the SN may reflect variability in the temporal trajectory of Parkinsonism during the clinical course of DLB. Despite abundant alpha‐synuclein‐immunoreactive structures in the LC and amygdala, the little involvement of the SN and neocortex suggests a pathological basis more closely associated with psychiatric manifestations than with motor symptoms or cognitive impairment. This selective vulnerability to LB pathology may contribute to the development of the predominant psychiatric symptoms in the prodromal stage of DLB. Further clinicopathological studies are warranted to clarify the role of LB pathology and support multidisciplinary approaches that integrate psychosomatic assessments with neuropathological investigations to evaluate late‐onset psychosis.

## AUTHOR CONTRIBUTIONS

Hiroshige Fujishiro performed microscopy analysis, data analysis, and drafted the manuscript. Ito Kawakami, Youta Torii, Shusei Arafuka, Shuji Iritani, and Kenji Ikeda participated in the study design and microscopic analysis. Kenichi Oshima organized the brain archives and selected the appropriate cases. Ito Kawakami, Kenichi Oshima, Youta Torii, Shusei Arafuka, Shuji Iritani, and Kenji Ikeda provided helpful advice on data interpretation and elaborated on the manuscript. All the authors have read and approved the final version of the manuscript.

## CONFLICT OF INTEREST STATEMENT

The authors declare no conflicts of interest.

## ETHICS APPROVAL STATEMENT

The personal information of patients and their bereaved families was protected. The Ethics Committee of the Tokyo Metropolitan Matsuzawa Hospital approved the use of this database. The study was conducted in accordance with the ethical standards of the 1964 Declaration of Helsinki and its subsequent amendments. Autopsies were performed in accordance with the “Corpse Autopsy and Preservation Law.”

## PATIENT CONSENT STATEMENT

After the patient died, written consent for publication was obtained from a proxy.

## CLINICAL TRIAL REGISTRATION

N/A.

## Data Availability

Data supporting this study are available from the corresponding author upon reasonable request.
